# Changes in clinical features and seasonal variations of Crohn’s disease at diagnosis: a 10-year observational study in China

**DOI:** 10.3389/fmed.2024.1489699

**Published:** 2024-11-06

**Authors:** Jun Deng, Yi Lu, Tao Liu, Min Zhang, Jia-yin Yao, Min Zhi

**Affiliations:** ^1^Department of Gastroenterology, The Sixth Affiliated Hospital, Sun Yat-sen University, Guangzhou, China; ^2^Guangdong Provincial Key Laboratory of Colorectal and Pelvic Floor Diseases, The Sixth Affiliated Hospital, Sun Yat-sen University, Guangzhou, China; ^3^Biomedical Innovation Center, The Sixth Affiliated Hospital, Sun Yat-sen University, Guangzhou, China; ^4^Department of Gastrointestinal Endoscopy, The Sixth Affiliated Hospital, Sun Yat-sen University, Guangzhou, China

**Keywords:** Crohn’s disease, disease phenotype, seasonal variation, medical therapy, changes

## Abstract

**Background and aims:**

The clinical aspects of Crohn’s disease (CD) at diagnosis determine its therapy and management. The onset of CD follows a seasonal pattern. We aimed to analyze changes in the clinical features and seasonal variations of newly CD patients over the last decade.

**Methods:**

CD patients were divided into cohort 1 (2012–2016) and cohort 2 (2017–2021). The clinical characteristics were collected and the trends according to the year and season of diagnosis were analyzed.

**Results:**

A total of 2038 patients were included. Cohort 1 had a considerably greater proportion of diarrhea, fever, hematochezia, weight loss and extraintestinal manifestations. The levels of platelet and C-reactive protein were higher in cohort 2 patients, but the opposite was true for albumin levels (*p*<0.05). The rate of increased eosinophils, increased gangliocyte and abundant lymphoplasmacytic infiltrate significantly decreased over the years. Patients with granulomas were diagnosed with CD at an earlier age (*p* = 0.006). Cohort 1 patients used more conventional drugs, while cohort 2 patients apply more biologics (*p*<0.05). The diagnosis occurred more frequently in summer and less frequently in winter. Patients diagnosed in winter had notably higher BMI, lower frequency of perianal disease and lowest incidence of asthenia and weight loss.

**Conclusion:**

The clinical phenotype, laboratory and pathological characteristics of CD has changed over time in China. The diagnosis of CD tends to have a seasonal trend with the highest incidence in summer. CD patients diagnosed in winter appear to have a milder form of the disease.

## 1 Introduction

Crohn’s disease (CD) is an idiopathic chronic inflammatory bowel disease (IBD) caused by a combination of environmental factors, genetic susceptibility and gut microbiota (1). According to current epidemiological studies, the global incidence of CD ranges from 0.1 to 16 per 100,000 people. The incidence is higher in industrialized countries than in developing countries (2). In recent years, CD has become more common in Asian countries such as Korea and Japan (3, 4). In China, CD is still a rare disease, although the incidence is increasing year by year. National epidemiological data on the incidence of CD are not yet available. The incidence of CD in urban China is 0.71 per 100,000 person-years in 2016 (5). Furthermore, the incidence of CD varies geographically, with a higher incidence in southern than in northern, as evidenced by an incidence rate of 1.09 per 100,000 person-years in Guangdong Province and 0.51 per 100,000 person-years in Daqing, Heilongjiang Province (6, 7).

At the time of diagnosis, patient demographics and disease behavioral aspects are thought to play an essential role in directing CD treatment and management (8). Differences in the epidemiology, genetic background and disease presentation of CD patients between Western and Asian countries have been reported (9, 10). In addition, the gender ratio of patients at diagnosis of CD is unusual across countries. In the United States, a slightly higher proportion of females than males are diagnosed with CD, whereas in Asian countries such as China, Japan and Korea, the proportion of males is higher than that of females (11–14). Due to ethnic variation, we need to investigate the demographic characteristics and disease profile of Asian CD patients at diagnosis. Furthermore, seasonal changes may influence the onset of IBD. It was discovered that the onset of IBD in children had a seasonal tendency, with a higher incidence in autumn and more complications in patients diagnosed in summer (15, 16). Data from a Japanese study suggested that adults with CD had a considerably greater incidence in the summer than in other seasons (17).

To date, investigations on the clinical characteristics of the CD population at diagnosis in China are still limited, particularly in terms of their temporal variation. Therefore, the aim of this study was to retrospectively analyze the clinical phenotypic, laboratory and pathological characteristics of newly diagnosed CD patients during the last 10 years and to describe the seasonal variation of disease manifestations at diagnosis. We were particularly interested in the temporal alterations of medical therapy and seasonal variations in the onset of CD.

## 2 Materials and methods

### 2.1 Study design and population

We retrospectively evaluated 3,083 consecutive CD patients attending the Sixth Hospital of Sun Yat-sen University (Guangzhou, China) from January 2012 to December 2021. The diagnosis of CD in children and adults was based on the Porto criteria and the criteria published by the European Crohn’s and Colitis Organization, respectively (18, 19). We collected the following information from patients at the time of CD diagnosis: demographic data, smoking and alcohol history, family history of IBD, clinical presentation, endoscopic findings, laboratory data, histological results, season of diagnosis and treatment medications. Data was collected from clinical records. Patients with incomplete clinical information or a stoma could not meet the inclusion criteria. In addition, patients with concomitant severe cardiac, pulmonary, neurological, mental, and other serious immunological disorders at the time of CD diagnosis were also excluded. Finally, we enrolled a total of 2038 CD patients. The study was reviewed and approved by the Ethics Committee of the Sixth Hospital of Sun Yat-sen University and an exemption from informed consent was obtained because of the retrospective study design.

### 2.2 Methods

The CD disease site and localization were identified using the Montreal classification (20). Disease sites were classified as L1 (ileum), L2 (colon), L3 (ileocolon). In addition, lesions involving only the upper gastrointestinal tract were considered as isolated L4 disease. Disease behavior could be categorized as B1 (nonrestrictive, non-penetrating), B2 (strict), or B3 (penetrating). Endoscopic evaluations were performed using the simple endoscopic score for CD (SES-CD) (21). We applied a modified version of SES-CD for an endoscopic evaluation of the small bowel, called the Simple Endoscopic Active Score for CD (SES-CDa) (22). The modified Rutgeerts’ score was used as endoscopic scoring system to assess the severity of recurrence of inflammation at the ileocolic anastomosis and in the neoterminal ileum (23). We conducted this study in Guangzhou, China, thus the seasons are defined as: spring (March, April and May), summer (June, July and August), autumn (September, October and November) and winter (December, January and February).

### 2.3 Statistical analysis

Continuous variables were expressed as the mean ± standard deviation (SD) if they followed normal distribution, and in case of non-normal distribution, median and range were used. They were analyzed by the independent sample *t*-test or the Mann–Whitney test. Categorical variables are described as counts with percentages and were evaluated using the chi-square test or Fisher’s exact test. Statistical significance was defined as a two-tailed *p* < 0.05. SPSS 22.0 software (IBM, Sommers, NY, USA) was used for statistical analysis. Image drawing was performed using GraphPad Prism 6 version 7.0 (GraphPad Software, USA).

## 3 Results

### 3.1 Patient characteristics

**Figure 1:**
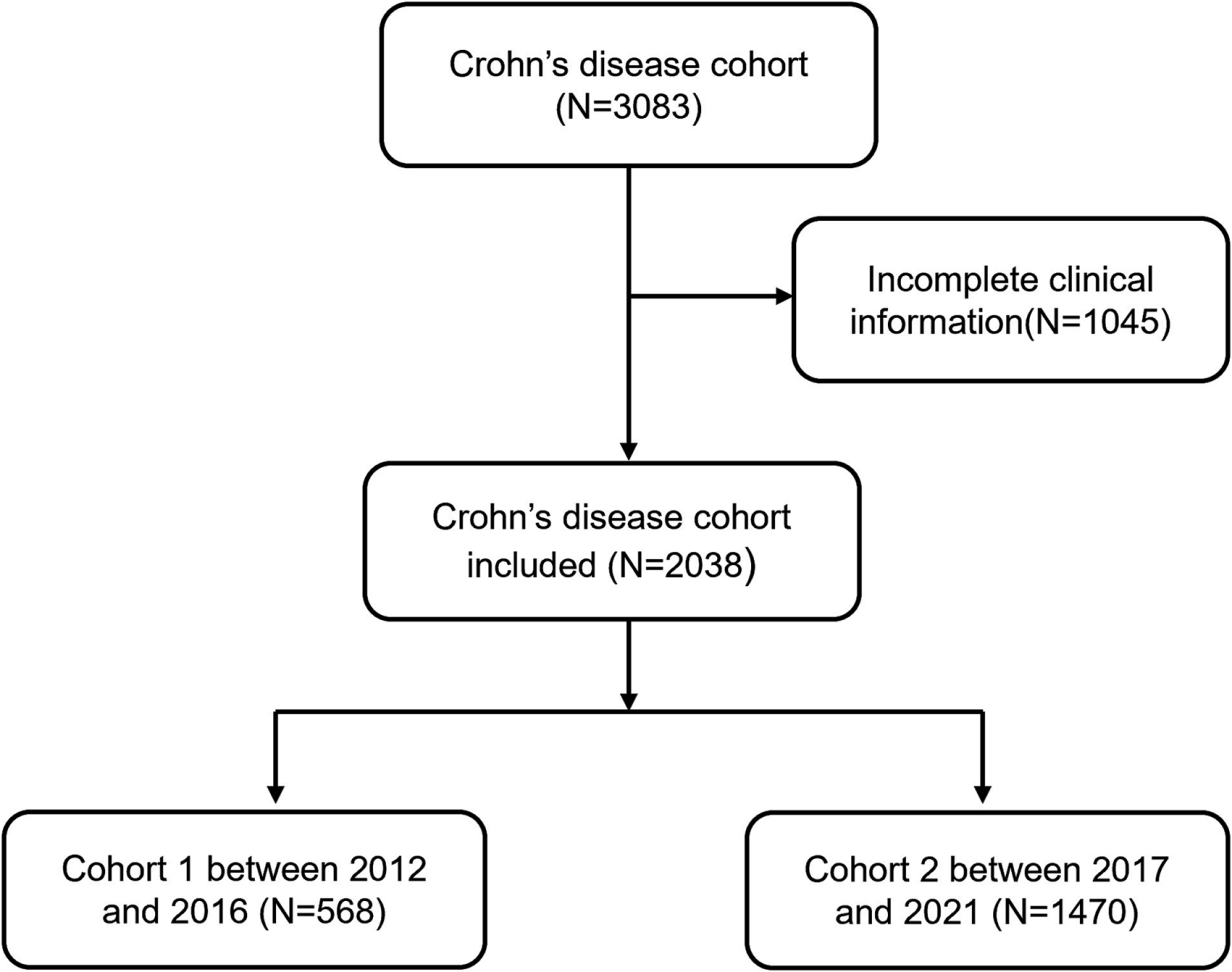
Flow diagram detailing study inclusion and exclusion.

In the recent decade, a total of 3,083 CD patients was newly diagnosed between January 2012 and December 2021. 1,045 patients (33.9%) were excluded because of incomplete information. Finally, 2038 patients with CD were enrolled in this study. We separated the patients into two groups: cohort 1 (diagnosed between 2012 and 2016) and cohort 2 (diagnosed between 2017 and 2021). Figure 1 displays the flowchart of the cohort. The baseline characteristics of the cohort 1 and cohort 2 are summarized in Table 1. 74% of newly diagnosed CD patients were male. The average age of CD patients at diagnosis was 28.23 ± 15.51 years. Only 8 patients (0.4%) had a family history of IBD. Cohort 1 showed more frequently smoking history and alcohol intake when compared with cohort 2 (7.7% vs. 4.8%, *p* = 0.009; 12.9% vs. 2.0%, *p*<0.001, respectively). Ileocolonic disease (L3, 70.1%) was the most common location at diagnosis, followed by colonic (L2, 16.0%) and ileal (L1, 8.6%) disease. A total of 1,368 patients (67.1%) displayed nonstricturing, nonpenetrating behavior at diagnosis. The percentage of perianal disease at diagnosis and perianal operation before diagnosis were not different between cohort 1 and cohort 2, nor was there a difference between perianal operation and intestinal resection at diagnosis. Cohort 2 had a lower proportion of intestinal resections before diagnosis than cohort 1 (11.0% vs. 14.3%, *p* = 0.043).

**Table 1:** Baseline characteristics of 2038 patients with CD.

	Overall	Cohort 1 (2012–2016)	Cohort 2 (2017–2021)	*p*-value^a^
Number of patients	2038	568	1,470	
Gender				
Male	1,059 (74.0)	431 (75.9)	1,509 (73.3)	0.26
Age at first IBD-related syptoms, yr.	24 (19–30)	23 (18–30)	24 (19–30)	0.224
Age at diagnosis, yr.	26 (21–33)	26 (20–33)	26 (21–33)	0.707
Family history of IBD	8 (0.4)	3 (0.5)	5 (0.3)	0.543
Smoking history	114 (5.6)	44 (7.7)	70 (4.8)	0.009^b^
History of alcohol intake	103 (5.1)	73 (12.9)	30 (2.0)	<0.001^b^
BMI	18.53 ± 3.06	18.34 ± 2.99	18.60 ± 3.09	0.083
Disease location at diagnosis				0.016^b^
L1	175 (8.6)	51 (9.0)	124 (8.4)	
L2	327 (16.0)	114 (20.1)	213 (14.5)	
L3	1,428 (70.1)	373 (65.7)	1,055 (71.8)	
Isolated L4	108 (5.3)	30 (5.3)	78 (5.3)	
Disease behavior at diagnosis				0.563
B1	1,368 (67.1)	393 (69.2)	975 (66.3)	
B2	346 (17.0)	87 (15.3)	259 (17.6)	
B3	284 (13.9)	76 (13.4)	208 (14.1)	
B2 + B3	40 (2.0)	12 (2.1)	40 (2.0)	
Perianal disease at diagnosis	1,282 (62.9)	336 (59.2)	946 (64.4)	0.074
History of perianal operation before diagnosis	550 (27.0)	167 (29.4)	383 (26.1)	0.127
Perianal operation at diagnosis	375 (18.4)	113 (19.9)	262 (17.8)	0.279
History of intestinal resection before diagnosis	243 (11.9)	81 (14.3)	162 (11.0)	0.043^b^
Intestinal resection at diagnosis	106 (5.2)	31 (5.5)	75 (5.1)	0.746
Seasons at diagnosis				0.035^b^
Spring	477 (23.4)	134 (23.6)	343 (23.3)	
Summer	742 (36.4)	183 (32.2)	559 (38.0)	
Autumn	509 (25.0)	164 (28.9)	345 (23.5)	
Winter	310 (15.2)	87 (15.3)	223 (15.2)	

Values are *n* (%), mean ± SD, or median (range).

a For the qualitative variables, chi-square test or Fisher’s exact test was used, and for quantitative variables Student *t* test or Mann–Whitney test was used, as appropriate.

b Statistical significance: *p* < 0.05.

### 3.2 Clinical symptoms

**Figure 2:**
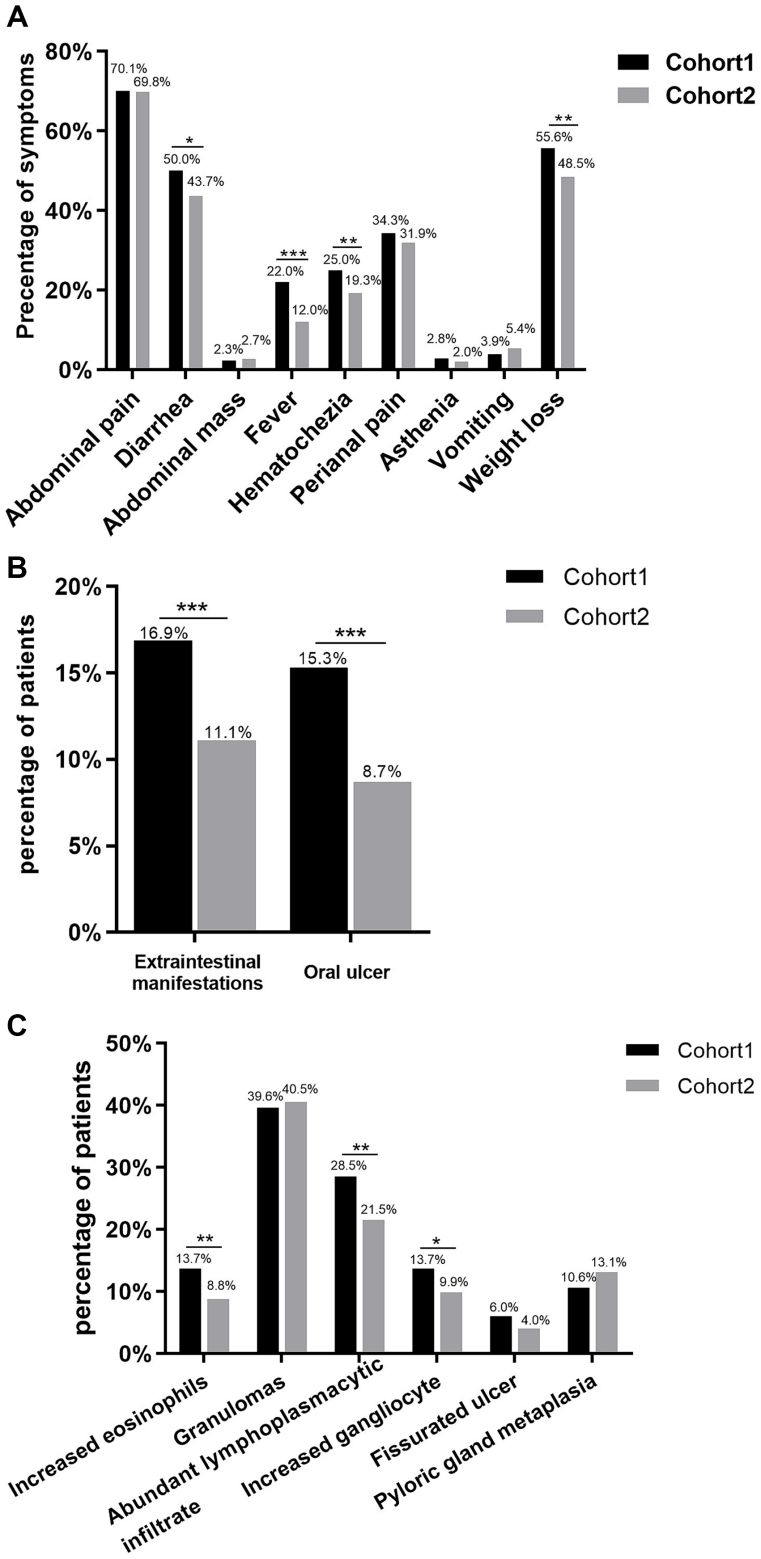
Clinical symptoms, extraintestinal manifestations, and histological characteristics of CD patients. **(A)** Comparing the percentage of clinical symptoms between cohort 1 and cohort 2. **(B)** Comparison of incidence of extraintestinal manifestations between cohort 1 and cohort 2. **(C)** Comparison of cohort 1 and cohort 2 histological characteristics. *p<0.05, **p<0.01, ***p<0.001.

As shown in Figure 2A, we counted the clinical symptoms commonly seen in CD patients at diagnosis, including abdominal pain, diarrhea, abdominal mass, fever, hematochezia, perianal pain, asthenia, vomiting, and weight loss. Compared with cohort 2, patients in cohort 1 had a larger proportion of symptoms of diarrhea, fever, hematochezia, and weight loss (50.0% vs. 43.7%, *p* = 0.01; 22.0% vs. 12.0%, *p*<0.001; 25.0% vs. 19.3%, *p* = 0.004; 55.6% vs. 48.5%, *p* = 0.004, respectively). We were equally interested in the incidence of extraintestinal manifestations and found that 12.71% of patients exhibited extraintestinal symptoms, with cohort 1 having a greater percentage than cohort 2 (16.9% vs. 11.1%, *p*<0.001, Figure 2B). We further found that the incidence of oral ulcers was higher in cohort 1 than in cohort 2 (15.3% vs. 8.7%, *p*<0.001, Figure 2B), whereas there were no significant differences in other extraintestinal manifestations such as arthralgia, erythema nodosum and skin rash (*p*>0.05).

### 3.3 Laboratory, endoscopic and histological characteristics

We analyzed laboratory indicators reflecting inflammatory activity and nutritional status, and discovered that the levels of platelet and C-reactive protein (CRP) were considerably greater in cohort 2 patients than in cohort 1 patients, although the opposite was true for albumin levels (Table 2). There was no statistical difference in SES-CD between cohort 1 and cohort 2. Furthermore, we investigated recurrence by comparing Rutgeerts scores in newly diagnosed patients with a history of intestinal resection and observed no difference in recurrence rates between the two cohorts (Table 2). The rate of increased eosinophils, increased gangliocytes and abundant lymphoplasmacytic infiltration has dramatically decreased over the years (Figure 2C). Patients with pathologically present granulomas were diagnosed with CD at a younger age than patients without granulomas (27.19 ± 9.97 vs. 28.93 ± 18.29, *p* = 0.006), but no significant differences were detected in the incidence of perianal disease, perianal operation and intestinal resection at diagnosis.

**Table 2:** Endoscopic evaluations, laboratory date and medical therapy of CD patients.

	Cohort 1 (2012–2016)	Cohort 2 (2017–2021)	*t*/*χ*2	*p*-value^a^
SES-CD	10.46 ± 6.85	10.39 ± 7.01	0.182	0.855
Rutgeerts score			0.046	0.831
i0-i2a	10 (37.0)	18 (34.6)		
i2b-i4	17 (63.0)	34 (65.4)		
Laboratory data				
Leukocyte	7.23 (5.52–9.42)	7.03 (5.63–8.95)	0.895	0.371
Hemoglobin	118 (102–132)	119 (101–133)	−1.255	0.210
Platelet	316 (260–405)	339 (270–426)	−3.288	0.001^b^
C-reactive protein	13.93 (5.69–35.93)	17.57 (5.39–45.82)	−4.289	<0.001^b^
Albumin	38.30 (34.18–42.65)	37.18 (28.98–41.09)	3.748	<0.001^b^
Medication use				
Oral 5-ASA	65 (11.4)	97 (6.6)	13.144	<0.001^b^
Systemic corticosteroids	154 (27.1)	359 (24.4)	1.575	0.209
Thiopurines	209 (36.8)	342 (23.3)	38.022	<0.001^b^
Anti-TNF agents	183 (32.2)	552 (37.6)	5.053	0.025^b^
Exclusive enteral nutrition	78 (13.7)	333 (22.7)	20.250	<0.001^b^
Thalidomide	33 (5.8)	54 (3.7)	4.576	0.032^b^
Methotrexate	8 (1.4)	28 (1.9)	0.582	0.446

Values are *n* (%), mean ± SD, or median (range).

a For the qualitative variables, chi-square test or Fisher’s exact test was used, and for quantitative variables Student *t* test or Mann–Whitney test was used, as appropriate.

b Statistical significance: *p* < 0.05.

### 3.4 Medical treatment

The medical therapy options for CD had changed dramatically over the last decade. As shown in Table 2, patients newly diagnosed with CD in the first 5 years were substantially more likely to be treated with 5-aminosalicylates (5-ASA), azathioprine, and thalidomide than those diagnosed in the second 5 years (11.4% vs. 6.6%, *p*<0.001; 36.8% vs. 23.3%, *p*<0.001; 5.8% vs. 3.7%, *p* = 0.032, respectively). However, the proportion of cohort 1 patients receiving anti-tumor necrosis factor (anti-TNF) agents was significantly lower than that of cohort 2 patients (32.3% vs. 37.6%, *p* = 0.025). Similar outcomes were also seen when comparing the percentage of patients in each group who received exclusive enteral nutrition (13.7% vs. 22.7%, *p*<0.001).

### 3.5 Seasonality of CD at diagnosis

**Figure 3:**
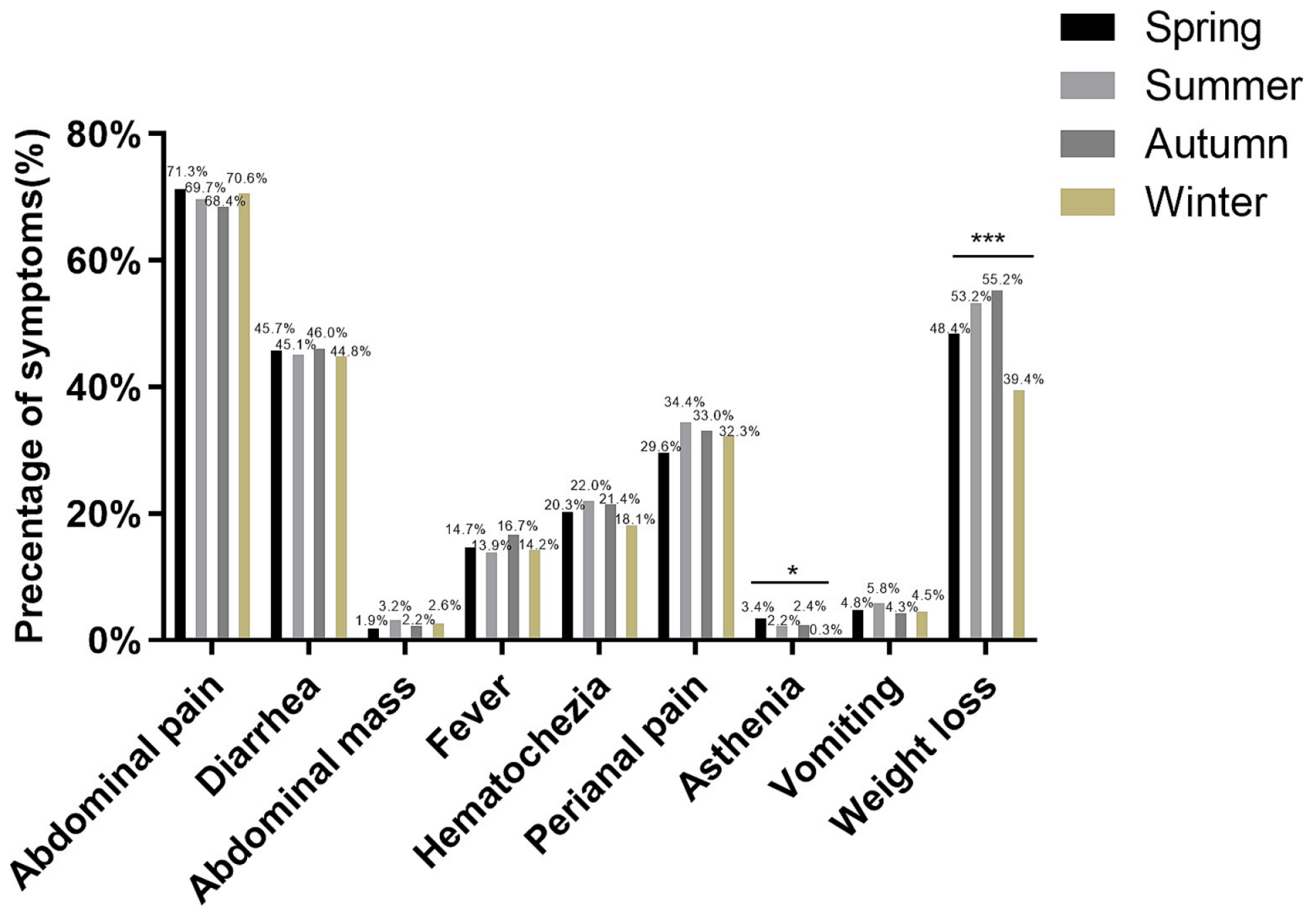
Clinical symptoms in patients with CD diagnosed in various seasons. *p<0.05, ***p<0.001.

We investigated the seasonal trend of CD when patients were diagnosed for the first time. The first CD diagnosis occurred more frequently in summer and less frequently in winter. Furthermore, we found significant variations in the seasonal distribution of cohort 1 and cohort 2 at diagnosis (*p* = 0.035, showed in Table 1). Therefore, we present the clinical data of patients based on the season of diagnosis in Table 3. BMI at CD diagnosis varied significantly among seasons (*p*<0.001). Patients had notably higher BMI at diagnosis in the winter than in other seasons. We discovered that the season had an effect on the frequency of perianal disease at diagnosis. Perianal disease was diagnosed less frequently in the winter [winter (56.5%), spring (61.6%), summer (66.8%), autumn (62.3%), *p* = 0.012]. In terms of clinical presentation, there were seasonal changes in asthenia and weight loss, with the lowest prevalence in winter (*p* = 0.045 and *p*<0.001, Figure 3). No seasonal variation was seen in extraintestinal manifestations. Due to the increasing frequency of biological treatment, we also analyzed the frequency of biological treatment in different seasons at diagnosis, and found that seasonal differences in biological application (*p* = 0.025), with the highest frequency of usage in summer (41.1%).

**Table 3:** Patient characteristics at diagnosis according to season.

	Spring	Summer	Autumn	Winter	*F*/*χ*2	*p*-value^a^
Number of patients	477	742	509	310	186.29	<0.001^b^
Gender					1.862	0.172
Male	344 (72.1)	547 (73.7)	365 (71.7)	253 (81.6)		
BMI	18.49 ± 3.14	18.36 ± 2.92	18.37 ± 3.01	19.22 ± 3.25	6.54	<0.001^b^
Disease location at diagnosis					6.466	0.693
L1	51 (10.7)	54 (7.3)	46 (9.0)	24 (7.7)		
L2	71 (14.9)	127 (17.1)	79 (15.5)	50 (16.1)		
L3	333 (69.8)	522 (70.3)	353 (69.4)	220 (71.0)		
Isolated L4	22 (4.6)	39 (5.3)	31 (6.1)	16 (5.2)		
Disease behavior at diagnosis					15.329	0.082
B1	325 (68.1)	523 (70.5)	325 (63.9)	195 (63.9)		
B2	73 (15.3)	119 (16.0)	92 (18.1)	62 (20.0)		
B3	67 (14.0)	86 (11.6)	68 (16.9)	45 (14.5)		
B2 + B3	12 (2.5)	14 (1.9)	6 (1.2)	8 (2.6)		
Perianal disease at diagnosis	294 (61.6)	496 (66.8)	317 (62.3)	175 (56.5)	10.887	0.012^b^
History of perianal operation before diagnosis	133 (28.9)	204 (27.5)	135 (26.5)	73 (23.5)	2.927	0.403
Perianal operation at diagnosis	89 (18.7)	140 (18.9)	92 (18.1)	54 (17.4)	0.364	0.948
History of intestinal resection before diagnosis	61 (12.8)	77 (10.4)	61 (12.0)	44 (14.2)	3.552	0.314
Intestinal resection at diagnosis	2 (0.6)	0 (0.0)	3 (0.6)	0 (0.0)	6.639	0.131

Values are *n* (%), mean ± SD.

a For the qualitative variables, chi-square test or Fisher’s exact test was used, and for quantitative variables Student *t* test or Mann–Whitney test was used, as appropriate.

b Statistical significance: *p* < 0.05.

## 4 Discussion

This is one of the largest studies to date describing disease phenotype of CD patients at diagnosis in China. The increasing tendency over the last few decades necessitates a greater focus on the characteristics of CD in newly developed Chinese population. However, Chinese studies analyzing the characteristics of CD patients are limited, and the changes in disease phenotype at diagnosis is unclear. In this study, we investigated the changes in clinical aspects of CD patients over the last decade for the first time. 74% of the 2038 CD patients were male. This revealed a higher male occurrence, which was consistent with the findings of previous Chinese and Asian studies but contradicted the findings of Western studies (5, 7, 11, 13, 14). In China, we found no significant change in the gender distribution of CD patients over time. The average age at of CD patients at diagnosis is similar between 2012 and 2021. Several studies have suggested that a positive family history in IBD patients ranges from 1.5 to 2.5% in Korea, 5 to 39% in the United States, and Europe falls somewhere in between (24–26). The positive family history of IBD in our data was 0.4%, and it did not grow over time. Because we included CD patients rather than IBD patients, our rate was lower than the results of other trails. Some specific risk factor exposures, such as smoking and alcohol intake, may promote the development of CD. We discovered that CD patients diagnosed between 2017 and 2021 had a considerably lower history of smoking and alcohol intake than those diagnosed between 2012 and 2016. This shows that Chinese patients may have gradually become more health conscious in recent years, rejecting poor lifestyle.

In addition to gender distribution, previous studies have indicated that clinical features of Asian CD patients also differ from those of Western CD patients in disease location and frequent perianal lesions at diagnosis (7, 14, 27). In Korea, approximately 64.7–68% of CD patients have ileocolonic disease, and 42.2–47% have perianal disease at diagnosis, whereas Western studies have found lower proportions of ileocolonic disease and perianal disease (28, 29). In our study, 70.1% of patients have ileocolonic disease and 62.9% have perianal disease, which is similar to the findings of other study in China (7) and the Korean studies mentioned above. The exact cause of the aforementioned variability is still unknown. Genetics, environmental variables, microbiome factors and other unrevealed reasons may have contributed to this phenomenon. The frequency of perianal disease in Chinese CD patients has increased over the last decade, although no significant difference exists.

The clinical manifestations of CD patient in China have also changed over time. In our data, the rate of diarrhea, fever, hematochezia and weight loss are significantly higher in the first 5 years than in the second 5 years between 2012 and 2021. Our results also reveal that extraintestinal manifestations occurring in 12.81% of patients, which is in line with other Asian reports (30, 31) and lower than Western studies (32, 33). In contrast to our findings, another prior Chinese study had reported the frequency of extraintestinal manifestations in CD patients was comparable to that of Western countries, at around 38% (7). We speculate that the reason for the discrepancy between the two studies is due to the fact that the prior study only comprised 48 patients, with the majority of them coming from Zhongshan, Guangdong. In contrast, our hospital is one of the largest IBD medical centers in China, and our patients come from all across the country. A relatively large number size of CD patients was enrolled in our study.

We next investigated changes in blood cell counts and biochemistry results and found that a large increase in platelet and CRP levels, as well as a significant decrease in albumin levels, among newly diagnosed CD patients over the last decade. These results indicate that the severity of CD patients seen at our IBD medical center is increasing year after year, implying that our center is becoming more influential in China. Microscopically, increased eosinophils, abundant lymphoplasmacytic infiltrate, granulomas, fissurated ulcer, pyloric gland metaplasia, cryptic architectural distortion and neural hyperplasia are frequent findings in CD (34–36). Although a granuloma has been used as a definitive criterion for the diagnosis of CD, its role in the pathophysiology and prognosis of CD remains unclear. The number of patients with increased eosinophils, abundant lymphoplasmacytic infiltrate and neural hyperplasia decreased dramatically over the decade, however the proportion of patients with granulomas did not change significantly in our study. We speculate that changes in specific environmental exposures over the last decade that could explain the considerable reductions in these pathological manifestations.

Since the 1990s, 5-ASA, corticosteroids and immunomodulators such as azathioprine/6-mercaptopurine have been widely used in the treatment of CD in China. Anti-TNF agents have become the mainstay of therapy in recent years due to increased availability and reimbursement by health insurance. We found that more newly diagnosed CD patients received anti-TNF therapy over the last decade, while fewer patients received 5-ASA and thiopurines. Additionally, the emergence of novel biologic agents, namely vedolizumab (VDZ) and ustekinumab (UST), has expanded medical options for disease control in China. However, they are not initially covered by health insurance, which limits the use of novel biologics. We do not display UST or VDZ data in this section.

Researchers have been interested in the seasonal variations in CD, and the findings are contradictory. Although studies from Japan and Italy have shown that the onset of CD is more likely in the summer (17, 37), another European investigation found no seasonality in disease onset (38). To the best of our knowledge, limited information is available on the seasonality of newly diagnosed CD in China. As a result, we investigated seasonal variations in CD diagnosis. We found that the proportion of identified CD was significantly higher in the summer than in other seasons. At present, no research findings can provide a clear explanation for the underlying mechanisms pertaining to IBD’s seasonality. The genesis and aggravation of CD are influenced by environmental factors. We believe temperature is one of the primary environmental elements and directly regulates the immune system. Inappropriate immune activation can trigger CD. We predicted that seasonal trends in CD diagnosis could be attributable to seasonal fluctuations in immunological responses, characterized by lower production of proinflammatory cytokines in the winter (39) and higher secretion of inflammatory mediators and leukocyte activity in the summer (40). Intestinal infections, such as enterovirus, salmonella and campylobacter infections, might peak in the summer, whereas respiratory infections are more likely in the autumn and winter. Seasonal exposure to these pathogenic pathogens may result in an immunological response that triggers intestinal inflammation. Intestinal infections and antibiotic use can also contribute to altered gut microbiota and damage to the intestinal mucosal, which can play an important role in the pathogenesis of CD (41, 42). These factors may account for the greater rate of CD diagnosis in the summer. In addition, there were seasonal variations in the incidence of perianal disease at diagnosis, with our data showing that summer is the most common season for perianal disease and winter is the least common season. The proportion of clinical symptoms also varies according to the season of diagnosis. Our results showed that the incidence of asthenia and weight loss is lowest incidence in the winter. This suggests a seasonal nature to the exacerbation of CD. CD patients diagnosed in the winter appear to have a milder form, whereas those diagnosed in the summer appear to have more sever disease. Our findings contradict other studies (17, 43). It is imperative to acknowledge that the seasonality of exacerbation may be influenced by the immune response and intestinal infections as previously described, but also by the seasonal secretion of corticoids (44), the seasonal habits or the cultural environment of each country (45). All of the mechanisms listed above are currently speculative. It would be worthwhile to explore the specific mechanisms by which seasonal changes contribute to the onset and development of CD. While we investigate the relationship between seasonal variations and CD diagnosis, the impact of geography on seasonal temperatures cannot be overlooked. Previous studies have linked latitude and geographical variations to risk of CD (46, 47). Our hospital is located in the subtropical city of Guangzhou in southern China, where the seasonal temperature difference varies less than in other parts of China. The origin of the patients was a key factor influencing the findings in this study. It should be emphasized that our hospital is known as one of the earliest and largest IBD centers in China. Patients visiting our center come from all around the country and are not confined to Guangdong province. The seasons in this study are defined according to the Chinese division of the four seasons. The authors believe that geographical considerations are not significant to the outcomes of this investigation.

The main strength of this study was the participation of over 2000 patients in China and the analysis of CD patients newly diagnosed in last 10 years, which could fill the knowledge gap regarding changes in disease phenotype and seasonal variations in CD patients. However, there are several limitations to this study. The first limitation is the retrospective and single-center design. Second, we must realize that the data presented here were not population-based. This was a hospital-based and cohort study. Therefore, the clinical manifestations of our research subjects may be relatively serious. Third, CDAI scores were lacking in some retrospectively collected data, so we failed to provide the comparison results of CDAI at diagnosis. CD is common among adolescents, the majority of whom are in school. This group of symptomatic patients may only visit the hospital to be diagnosed with Crohn’s disease over the extended summer vacation. The effect of enrolled patients’ occupation and age on seasonal fluctuation in disease diagnosis was not investigated in our study. This is an additional limitation of this study.

## 5 Conclusion

In conclusion, our study revealed that the clinical phenotypic, laboratory and pathological characteristics of CD patients newly diagnosed in China has changed over the last decade. The diagnosis of CD tends to have a seasonal tendency with the highest incidence in the summer. CD patients diagnosed in the winter appear to have a milder form of the disease.

## Data Availability

The raw data supporting the conclusions of this article will be made available by the authors, without undue reservation.

## References

[1] Torres J Mehandru S Colombel JF Peyrin-Biroulet L. Crohn's disease. Lancet. (2017) 389:1741–55. doi: 10.1016/S0140-6736(16)31711-1 27914655

[2] Ng SC Shi HY Hamidi N Underwood FE Tang W Benchimol EI. Worldwide incidence and prevalence of inflammatory bowel disease in the 21st century: a systematic review of population-based studies. Lancet. (2017) 390:2769–78. doi: 10.1016/S0140-6736(17)32448-0, PMID: 29050646

[3] Park SH Kim YJ Rhee KH Kim YH Hong SN Kim KH. A 30-year trend analysis in the epidemiology of inflammatory bowel disease in the Songpa-Kangdong District of Seoul, Korea in 1986-2015. J Crohns Colitis. (2019) 13:1410–7. doi: 10.1093/ecco-jcc/jjz081, PMID: 30989166

[4] Murakami Y Nishiwaki Y Oba MS Asakura K Ohfuji S Fukushima W. Estimated prevalence of ulcerative colitis and Crohn's disease in Japan in 2014: an analysis of a nationwide survey. J Gastroenterol. (2019) 54:1070–7. doi: 10.1007/s00535-019-01603-8, PMID: 31309327

[5] Xu L He B Sun Y Li J Shen P Hu L. Incidence of inflammatory bowel disease in urban China: a Nationwide population-based study. Clin Gastroenterol Hepatol. (2023) 21:3379–3386.e29. doi: 10.1016/j.cgh.2023.08.013, PMID: 37660767

[6] Yang H Li Y Wu W Sun Q Zhang Y Zhao W. The incidence of inflammatory bowel disease in northern China: a prospective population-based study. PLoS One. (2014) 9:e101296. doi: 10.1371/journal.pone.0101296, PMID: 25029440 PMC4100738

[7] Zeng Z Zhu Z Yang Y Ruan W Peng X Su Y. Incidence and clinical characteristics of inflammatory bowel disease in a developed region of Guangdong Province, China: a prospective population-based study. J Gastroenterol Hepatol. (2013) 28:1148–53. doi: 10.1111/jgh.12164, PMID: 23432198

[8] Barnes EL Loftus EV Jr Kappelman MD. Effects of race and ethnicity on diagnosis and management of inflammatory bowel diseases. Gastroenterology. (2021) 160:677–89. doi: 10.1053/j.gastro.2020.08.064, PMID: 33098884

[9] Liu JZ van Sommeren S Huang H Ng SC Alberts R Takahashi A. Association analyses identify 38 susceptibility loci for inflammatory bowel disease and highlight shared genetic risk across populations. Nat Genet. (2015) 47:979–86. doi: 10.1038/ng.3359, PMID: 26192919 PMC4881818

[10] Shi HY Levy AN Trivedi HD Chan FKL Ng SC Ananthakrishnan AN. Ethnicity influences phenotype and outcomes in inflammatory bowel disease: a systematic review and Meta-analysis of population-based studies. Clin Gastroenterol Hepatol. (2018) 16:190–197.e11. doi: 10.1016/j.cgh.2017.05.047, PMID: 28603049 PMC5722715

[11] Ng SC Tang W Ching JY Wong M Chow CM Hui AJ. Incidence and phenotype of inflammatory bowel disease based on results from the Asia-Pacific Crohn's and colitis epidemiology study. Gastroenterology. (2013) 145:158–165.e2. doi: 10.1053/j.gastro.2013.04.007, PMID: 23583432

[12] Zhao J Ng SC Lei Y Yi F Li J Yu L. First prospective, population-based inflammatory bowel disease incidence study in mainland of China: the emergence of "western" disease. Inflamm Bowel Dis. (2013) 19:1839–45. doi: 10.1097/MIB.0b013e31828a6551, PMID: 23669403

[13] Shah SC Khalili H Gower-Rousseau C Olen O Benchimol EI Lynge E. Sex-based differences in incidence of inflammatory bowel diseases-pooled analysis of population-based studies from Western countries. Gastroenterology. (2018) 155:1079–1089.e3. doi: 10.1053/j.gastro.2018.06.043, PMID: 29958857

[14] Yang SK. How does the epidemiology of inflammatory bowel disease differ between east and west? A Korean perspective. Inflamm Intest Dis. (2017) 2:95–101. doi: 10.1159/000454712, PMID: 30018960 PMC5988201

[15] Dharmaraj R Jaber A Arora R Hagglund K Lyons H. Seasonal variations in onset and exacerbation of inflammatory bowel diseases in children. BMC Res Notes. (2015) 8:696. doi: 10.1186/s13104-015-1702-y, PMID: 26588900 PMC4654892

[16] Glapa-Nowak A Szczepanik M Kwiecien J Szaflarska-Poplawska A Flak-Wancerz A Iwanczak B. Insolation and disease severity in Paediatric inflammatory bowel disease-a multi-Centre cross-sectional study. J Clin Med. (2020) 9:3957. doi: 10.3390/jcm9123957, PMID: 33297324 PMC7762204

[17] Araki M Shinzaki S Yamada T Arimitsu S Komori M Shibukawa N. Age at onset is associated with the seasonal pattern of onset and exacerbation in inflammatory bowel disease. J Gastroenterol. (2017) 52:1149–57. doi: 10.1007/s00535-017-1313-6, PMID: 28168321

[18] IBD Working Group of the European Society for Paediatric Gastroenterology, Hepatology and Nutrition. Inflammatory bowel disease in children and adolescents: recommendations for diagnosis‐the Porto criteria. J Pediatr Gastroenterol Nutr. (2005) 41:1–7. doi: 10.1097/01.mpg.0000163736.30261.82, PMID: 15990620

[19] Gomollón F Dignass A Annese V Tilg H van Assche G Lindsay JO. 3rd European evidence-based consensus on the diagnosis and Management of Crohn’s disease 2016: part 1: diagnosis and medical management. J Crohns Colitis. (2017) 11:3–25. doi: 10.1093/ecco-jcc/jjw168, PMID: 27660341

[20] Silverberg MS Satsangi J Ahmad T Arnott ID Bernstein CN Brant SR. Toward an integrated clinical, molecular and serological classification of inflammatory bowel disease: report of a working party of the 2005 Montreal world congress of gastroenterology. Can J Gastroenterol. (2005) 19:5A–36A. doi: 10.1155/2005/269076, PMID: 16151544

[21] Daperno M D'Haens G Van Assche G Baert F Bulois P Maunoury V. Development and validation of a new, simplified endoscopic activity score for Crohn's disease: the SES-CD. Gastrointest Endosc. (2004) 60:505–12. doi: 10.1016/s0016-5107(04)01878-4, PMID: 15472670

[22] Takenaka K Ohtsuka K Kitazume Y Matsuoka K Nagahori M Fujii T. Utility of magnetic resonance Enterography for small bowel endoscopic healing in patients with Crohn's disease. Am J Gastroenterol. (2018) 113:283–94. doi: 10.1038/ajg.2017.464, PMID: 29257147

[23] Lopes S Andrade P Afonso J Rodrigues-Pinto E Dias CC Macedo G. Correlation between calprotectin and modified Rutgeerts score. Inflamm Bowel Dis. (2016) 22:2173–81. doi: 10.1097/MIB.0000000000000850, PMID: 27482974

[24] Park JB Yang SK Byeon JS Park ER Moon G Myung SJ. Familial occurrence of inflammatory bowel disease in Korea. Inflamm Bowel Dis. (2006) 12:1146–51. doi: 10.1097/01.mib.0000235094.01608.59 17119389

[25] Wang PQ Hu J Al Kazzi ES Akhuemonkhan E Zhi M Gao X. Family history and disease outcomes in patients with Crohn's disease: a comparison between China and the United States. World J Gastrointest Pharmacol Ther. (2016) 7:556–63. doi: 10.4292/wjgpt.v7.i4.556, PMID: 27867689 PMC5095575

[26] Roma ES Panayiotou J Pachoula J Constantinidou C Polyzos A Zellos A. Inflammatory bowel disease in children: the role of a positive family history. Eur J Gastroenterol Hepatol. (2010) 22:1–5. doi: 10.1097/MEG.0b013e32832e2bd8, PMID: 19543100

[27] Kim HJ Hann HJ Hong SN Kim KH Ahn IM Song JY. Incidence and natural course of inflammatory bowel disease in Korea, 2006-2012: a nationwide population-based study. Inflamm Bowel Dis. (2015) 21:623–30. doi: 10.1097/MIB.0000000000000313, PMID: 25647154

[28] Park SH Yang SK Park SK Kim JW Yang DH Jung KW. Long-term prognosis of crohn's disease and its temporal change between 1981 and 2012: a hospital-based cohort study from Korea. Inflamm Bowel Dis. (2014) 20:488–94. doi: 10.1097/01.MIB.0000441203.56196.46, PMID: 24412992

[29] Hong SW Ye BD Cheon JH Lee JH Koo JS Jang BI. Clinical features and long-term prognosis of Crohn's disease in Korea: results from the prospective CONNECT study. Gut Liver. (2022) 16:907–20. doi: 10.5009/gnl210299, PMID: 35321956 PMC9668509

[30] Jiang L Xia B Li J Ye M Yan W Deng C. Retrospective survey of 452 patients with inflammatory bowel disease in Wuhan city, Central China. Inflamm Bowel Dis. (2006) 12:212–7. doi: 10.1097/01.MIB.0000201098.26450.ae 16534423

[31] Ohta Y Taida T Kato J Ogasawara S Oyama Y Mamiya Y. Clinical features focusing on Extraintestinal manifestations in Japanese patients with inflammatory bowel diseases: Far East 1000. Digestion. (2023) 104:328–34. doi: 10.1159/000529816, PMID: 36893744

[32] Mendoza JL Lana R Taxonera C Alba C Izquierdo S Diaz-Rubio M. Extraintestinal manifestations in inflammatory bowel disease: differences between Crohn's disease and ulcerative colitis. Med Clin. (2005) 125:297–300. doi: 10.1157/13078423, PMID: 16159555

[33] Rogler G Singh A Kavanaugh A Rubin DT. Extraintestinal manifestations of inflammatory bowel disease: current concepts, treatment, and implications for disease management. Gastroenterology. (2021) 161:1118–32. doi: 10.1053/j.gastro.2021.07.042, PMID: 34358489 PMC8564770

[34] Kumarasinghe MP Quek TP Chau CY Mustapha NR Luman W Ooi CJ. Endoscopic biopsy features and diagnostic challenges of adult Crohn's disease at initial presentation. Pathology. (2010) 42:131–7. doi: 10.3109/00313020903494979, PMID: 20085514

[35] Villanacci V Antonelli E Reboldi G Salemme M Casella G Bassotti G. Endoscopic biopsy samples of naive "colitides" patients: role of basal plasmacytosis. J Crohns Colitis. (2014) 8:1438–43. doi: 10.1016/j.crohns.2014.05.003, PMID: 24931895

[36] Sankey EA Dhillon AP Anthony A Wakefield AJ Sim R More L. Early mucosal changes in Crohn's disease. Gut. (1993) 34:375–81. doi: 10.1136/gut.34.3.375, PMID: 8472987 PMC1374145

[37] Aratari A Papi C Galletti B Angelucci E Viscido A D’Ovidio V. Seasonal variations in onset of symptoms in Crohn's disease. Dig Liver Dis. (2006) 38:319–23. doi: 10.1016/j.dld.2005.10.002, PMID: 16289974

[38] Romberg-Camps MJ Hesselink-van de Kruijs MA Schouten LJ Dagnelie PC Limonard CB Kester AD. Inflammatory bowel disease in South Limburg (the Netherlands) 1991-2002: incidence, diagnostic delay, and seasonal variations in onset of symptoms. J Crohns Colitis. (2009) 3:115–24. doi: 10.1016/j.crohns.2008.12.002, PMID: 21172254

[39] Nelson RJ. Seasonal immune function and sickness responses. Trends Immunol. (2004) 25:187–92. doi: 10.1016/j.it.2004.02.001, PMID: 15039045

[40] Linden M Larson M Prellner T Brattsand R Laitinen LA. Seasonal variation in the function of blood monocytes obtained from healthy nonsmokers, asymptomatic smokers, and smokers with chronic bronchitis. Chronobiol Int. (1994) 11:266–72. doi: 10.3109/07420529409067794, PMID: 7954908

[41] Mark-Christensen A Lange A Erichsen R Froslev T Esen BO Sorensen HT. Early-life exposure to antibiotics and risk for Crohn's disease: a Nationwide Danish birth cohort study. Inflamm Bowel Dis. (2022) 28:415–22. doi: 10.1093/ibd/izab085, PMID: 34000050 PMC8889299

[42] Narula N Wong ECL Pray C Marshall JK Rangarajan S Islam S. Yusuf S. Associations of antibiotics, hormonal therapies, Oral contraceptives, and long-term NSAIDS with inflammatory bowel disease: results from the prospective urban rural epidemiology (PURE) study. Clin Gastroenterol Hepatol. (2023) 21:2649–2659.e16. doi: 10.1016/j.cgh.2022.11.037, PMID: 36528284

[43] Jung YS Song CS Kim ER Park DI Kim YH Cha JM. Seasonal variation in months of birth and symptom flares in Korean patients with inflammatory bowel disease. Gut Liver. (2013) 7:661–7. doi: 10.5009/gnl.2013.7.6.661, PMID: 24312706 PMC3848533

[44] Matchock RL Dorn LD Susman EJ. Diurnal and seasonal cortisol, testosterone, and DHEA rhythms in boys and girls during puberty. Chronobiol Int. (2007) 24:969–90. doi: 10.1080/07420520701649471, PMID: 17994350

[45] Neamti L Drugan TC Drugan C Silaghi C Ciobanu L Ilyes T. Assessing seasonal variations of biomarkers in inflammatory bowel disease. Eur J Gastroenterol Hepatol. (2024) 36:993–9. doi: 10.1097/MEG.0000000000002795, PMID: 38973542

[46] Armitage EL Aldhous MC Anderson N Drummond HE Riemersma RA Ghosh S. Incidence of juvenile-onset Crohn's disease in Scotland: association with northern latitude and affluence. Gastroenterology. (2004) 127:1051–7. doi: 10.1053/j.gastro.2004.06.024, PMID: 15480983

[47] Nerich V Monnet E Etienne A Louafi S Ramee C Rican S. Geographical variations of inflammatory bowel disease in France: a study based on national health insurance data. Inflamm Bowel Dis. (2006) 12:218–26. doi: 10.1097/01.MIB.0000206540.38834.8c, PMID: 16534424

